# The ecological services of plant communities in parks for climate control and recreation—A case study in Shanghai, China

**DOI:** 10.1371/journal.pone.0196445

**Published:** 2018-04-25

**Authors:** Zhigang Li, Dan Chen, Shize Cai, Shengquan Che

**Affiliations:** 1 Department of Landscape Architecture, School of Agriculture and Biology, Shanghai Jiao Tong University, Shanghai, China; 2 Key Laboratory of Urban Agriculture (South) Ministry of Agriculture, Shanghai Jiao Tong University, Shanghai, China; Public Library of Science, UNITED KINGDOM

## Abstract

Mitigating extreme heat in urban areas is beneficial and sometimes critical to human health. Thriving plant communities in community parks play an important role in mitigating extreme heat through providing cooling effect, while inevitably affecting how people perceive the benefits of using community parks for recreation. Thus, the impacts of plant communities on the thermal environment should be quantified to determine the optimal structure of the plant community. The goal would be to harmonize the functions of improving the thermal environment with the preferences people have related to the recreational benefits of plant communities with various levels of vegetation density. In this paper, the correlations between the structural characteristics of plant communities and their function in mitigating the thermal environment were investigated on calm summer days in Xincheng Central Park, Minhang District, Shanghai, China. In addition to analyzing the plant communities present and their effects on the park microclimate, a questionnaire was employed to determine the plant community preferences of recreational park users. The results showed that plant communities could reduce the air temperature by 1.23–2.42 °C and increase the relative humidity by 2.4–4.2% during the daytime. The microclimate conditions in plant communities with varying vegetation densities were significantly different. The canopy density and leaf area index primarily controlled the temperature reduction, while the canopy density and total canopy cover ratio primarily controlled the increase in humidity; meanwhile, these correlations varied at different times of the day. Moreover, most of the park users preferred a moderately dense plant community which met their environmental perceptions for recreation in parks. Age or education level variables of park users would also predict preferences for different plant community densities. Ultimately, one plant community pattern with appropriate canopy density (60%), leaf area index (≥3) and canopy cover ratio (total 0.80–1.20, with 0.6–0.75 for trees and 0.20–0.45 for shrubs/woodland area) was recommended, which would harmonize the functions of the mitigation of the thermal environment with most people’s perception of a desirable vegetation density.

## Introduction

As urban populations grow and expand, the creation of urban heat islands (UHIs) have been aggravated mainly by the creation of impervious ground surfaces, the loss of vegetation, and generation of anthropogenic heat that is released from industrial and of human activities [1–4]. Many studies have been conducted, confirming that in addition to increasing air and surface temperatures in urban areas, UHIs can result in increased water and energy consumption for air conditioning as well as an increase in the concentration of ground level pollutants; these changes influence the habitability of cities and can even lead to direct or indirect harm to human health [5–8]. Considering the enormous adverse impacts caused by UHIs, the research community has conducted numerous research projects in the field to determine how to improve the urban thermal environment.

Among all possible cooling measures, vegetation provides one of the most effective strategies used to mitigate the UHI effect directly through shading and indirectly through evapotranspiration [9–15]. Numerous studies have been conducted to measure and evaluate the ability of vegetation to regulate the microclimate of urban areas [12, 16, 17], to adapt to patterns of climate change [13], to reduce energy use [18], and to improve the comfort of the urban population [19, 20]. Some related studies have reported that air temperature in the shade of trees would be significantly lower than in areas without trees during summer. Shashua-Bar et al. (2010) found that the grass planting combined with shade trees could make pronounced effects on air temperature reduction [15]. By referring to the previous studies, it was further found that structural characteristics of plant communities were important factors for mitigating the thermal environment [21, 22]. Zhang et al. (2013) investigated the interrelations between the structural characteristics of plant community and microclimate conditions, which testified canopy density, canopy area, tree height and solar radiation as the main factors influencing the cooling and humidifying effects from plant communities significantly [22].

Plant communities in urban parks could not only mitigate the thermal environment, but also provide the chances for urban residents to get close to nature [23]. Urban residents often expect to make frequent visits to parks close to home, which generate significant improvements in the perceived physical and psychological benefits of people as well as the well-being for users during periods of heat stress [24]. The attractiveness of urban parks to residents could be influenced by many factors including the structure features of the plant communities, the locomotor abilities of urban residents, and the desire of park users to observe wildlife etc. [23, 25]. Generally, researchers have found that park visitors have a strong preference for plant community scenes that are characterized by a moderate degree of openness along with high degrees of smoothness of ground texture which improves accessibility [26, 27]. From the perspective of landscape ecology, Parsons noted that while densely planted forest patches supported a diversity of wildlife habitats, people tended to prefer more open grassy areas punctuated by occasional groupings of trees and shrubs, i.e., moderately open settings [28]. Furthermore, residents with different socio-demographic characteristics may prefer different plant community scenes that vary in vegetation density [29–31]. In past studies, age was the most significant explanatory variable with the desire for a natural state in a forest decreasing with increasing age [23, 32, 33].

The structural characteristics of plant communities not only affect ability of the community to mitigate thermal environment but also influence the perceived appropriateness of park areas for outdoor recreation. However, previous studies mainly focused on quantifying the cooling and humidifying effects from plant communities or on comparing the preference of people for plant communities with different vegetation densities. Nevertheless, these studies did not consider the microclimate control and environmental perception of plant community together, especially on hot summer days.

The present study was designed to both measure the climate control service provided by plant communities in a park and document the structural nature of these plant communities. The study simultaneously employed a questionnaire survey to determine the plant communities that park visitors prefer to enjoy when using the park for recreation such as walking, chatting, and dancing, etc. In the present study, we aimed to 1) find whether there was a significant difference in the microclimate conditions of plant communities varying in vegetation density; 2) quantify the ability of vegetation structure features on diurnal variation in cooling and humidifying effects; 3) survey the plant community scenes that most respondents preferred for outdoor recreation in urban parks; 4) recommend a plant community structure with high cooling ecosystem service on hot summer days that could also meet most respondents’ environmental perceptions of a desirable vegetation density for outdoor recreation. We selected the research sites in a community park in Shanghai where the surrounding air temperature often exceeds 35 °C in summer. This oppressive heat will often limit the outdoor recreation activities of park users. However, by providing plant communities that could effectively improve the microclimate of the park, land managers could provide relatively open fields at slightly cooler temperatures for potential outdoor recreation activities. This information will help land managers to understand the role of plant communities on mitigating UHIs in urban areas, and specifically, help to improve the thermal and recreational environments through improved design of the structural characteristics of plant communities.

## Materials and methods

### Ethics statement

The field studies were conducted in a public park of Shanghai city where specific permits were not required to the experiments conduct. These field experiments did not involve any endangered or protected species. Shanghai Jiao Tong University and the Shanghai Urban Planning and Land Resources Bureau approved the questionnaire survey. All the contents in the questionnaire were not health-related and did not involve clinical research. The questionnaires were used anonymously, and did not cause any difficulties or adverse effects to the participants. Before the survey, the respondents were informed on the purpose of this investigation, ensuring the confidentiality of their answers. When the participants agreed to fill in questionnaires and checked the box stating their agreement, the interview began.

### Description of the study area

Studies were conducted in Shanghai, China (30°82′30″–31°82′70″N and 120°85′20″–121°84′50″E), a city with a total population of 23.0 million. Shanghai experiences a subtropical oceanic monsoon climate with hot summers and cold winters. Based on meteorological data collected from 1952 to 2015, the Meteorological Bureau of Shanghai has recorded a minimum monthly mean air temperature of 4 C in January, and a maximum of 28 °C in July and August. The annual average precipitation was approximately 1200 mm, approximately 60% of which occurred from May to September.

**Fig 1:**
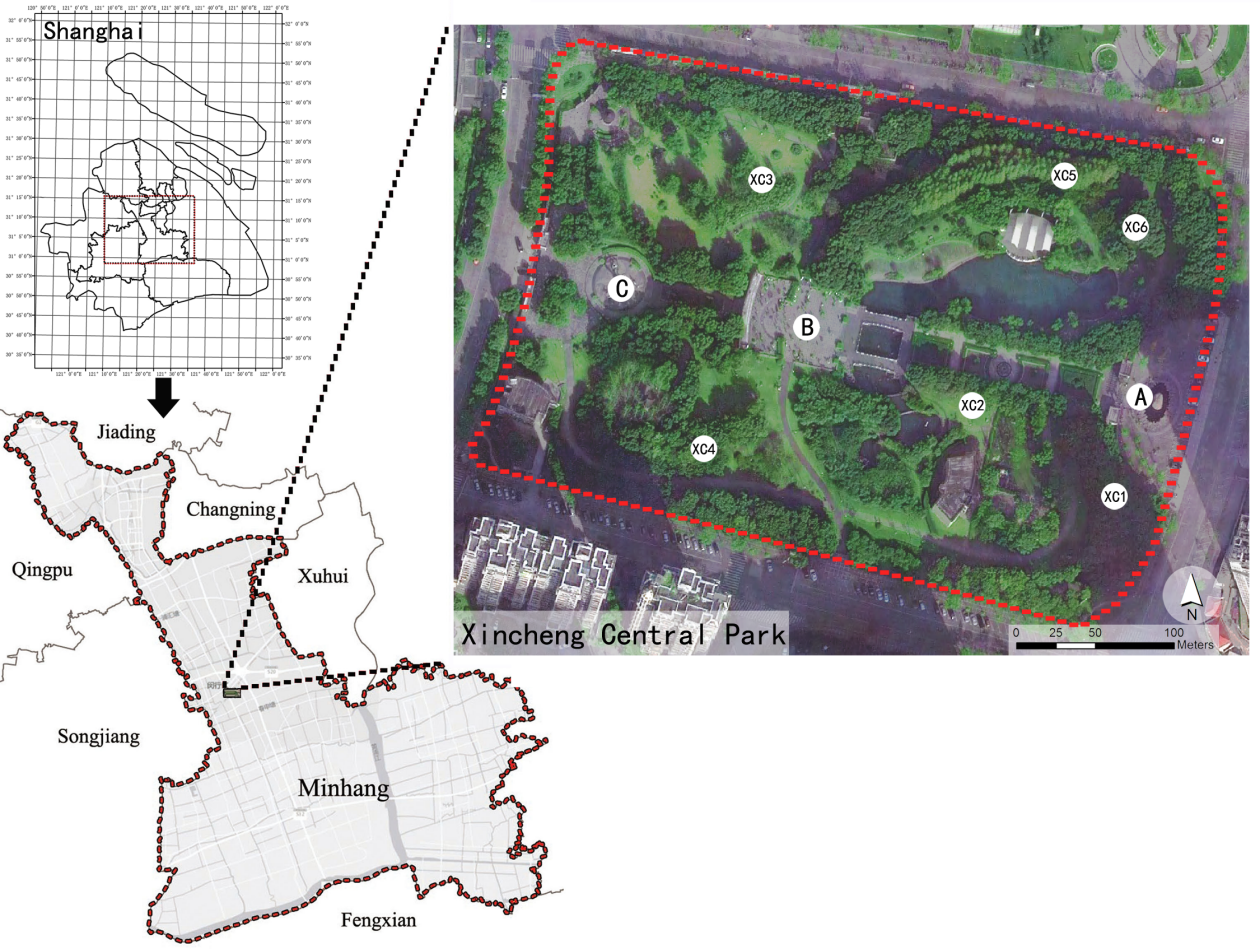
A map of the study area and measurement sites in Xincheng Central Park, Shanghai, China. XC1 to XC6 represent the six selected plant community sites used for the investigation, while the capital letters represent the three control plots in this park. Datasource: MAP WORLD 2016. http://www.tianditu.com/.

The present study was conducted in Xincheng Central Park, a community park covering 4.3 ha and located in the Minhang District in southwestern Shanghai (**Fig 1**). This site was chosen because 1) this community park can be regarded as a typical area currently experiencing urbanization in Shanghai, and is located in the suburbs; 2) this very level park has an average elevation of less than 10 m; therefore, temperature differences caused by topography are negligible and can be ignored; 3) densely spaced residential buildings and some commercial structures surround the park, and most residents visit this site for recreational activities including walking, chatting, and dancing, etc.; 4) approximately 90% of the total area is covered by vegetation, with different vegetation densities from very dense, especially the dense plant communities, to quite open; 5) compared with other community parks in the center of Shanghai or in new town, plants in this park were planted in 1990–1995, and consequently the plant communities have now stabilized.

### Summary of field work

Field work for this study included two primary aspects, a series of field studies related to plant community and microclimate as well as a questionnaire. The field studies mainly included the identification and investigation of plant communities varying in vegetation density as well as the measurement of air temperature and relative humidity. The purpose of the field studies was to compare the microclimate in plant communities with varying vegetation densities and to evaluate the ability of structural characteristics to explain the variation of cooling and humidifying effects from plant communities. While the questionnaire survey focused on identifying the potential plant community scenes that met most respondents’ environmental perceptions for outdoor recreation in park areas.

#### Identification of plant community density

First, variations in plant community density needed to be classified. Based on the prevailing vegetation density in urban plant communities of Shanghai and referring to Zhang Hua’s research [34], photographs were initially taken of the plant communities at several sites within Xincheng Central Park. Then, the differences in vegetation density of the park’s plant communities were identified using a consensus of the visual perception of our research team members. Plant communities were categorized into three grades of vegetation density: open, moderately dense, and dense. In the present study, the canopy cover ratio (proportion of canopy cover for trees and shrubs/woodland area in each target plant community, CC) was measured and recorded as one further quantitative indicator of vegetation density [34].

#### Selection and investigation of plant communities

After the identification of three levels of plant community density, six typical small-scale plant communities with different combinations of trees, shrubs and grasses were chosen for the present study. That is, two sites for were selected for open (sites XC2 and XC3), moderately dense (sites XC1 and XC6), and dense plant communities (XC4 and XC5; **Table 1, Fig 1**). Each plant community occupied 500–600 m^2^ of land and these communities were uniformly distributed in this park (**Fig 1**). During the selection of these target sites, plant communities with steep slopes, which would complicate the field investigation and microclimate measurement, were eliminated. And the selected plant communities were chosen to be located 10 m away from hills and water bodies for avoiding the effects of surrounding elements on the experimental data. Furthermore, the areas within each selected plant community had similar or uniform structural characteristics within each community type, and they could be distinguished from surrounding plant groups. In addition, three unshaded paved squares in this park were selected as control plots with areas of 400–600 m^2^ each (**Fig 1)**.

**Table 1:** The structural characteristics of six plant communities in Xincheng Central Park, Shanghai, China.

Code	Vegetation density	CD (%)	LAI	Total CC	H (m)	DBH (cm)
**XC1**	Moderately dense	72.51	3.575	1.10	9.39	16.80
**XC2**	Open	34.87	2.227	0.60	5.17	18.83
**XC3**	Open	45.24	2.373	0.55	12.00	40.00
**XC4**	Dense	66.73	2.976	1.25	10.66	15.62
**XC5**	Dense	72.98	3.648	1.40	7.83	13.36
**XC6**	Moderately dense	76.48	3.896	1.15	5.88	16.17

Note: Total CC, total canopy cover ratio; CD, canopy density; DBH, mean diameter at breast height of the trees; H, mean height of the trees; LAI, leaf area index; XC1 to XC6, plant community site numbers in Xincheng Central Park.

**Fig 2:**
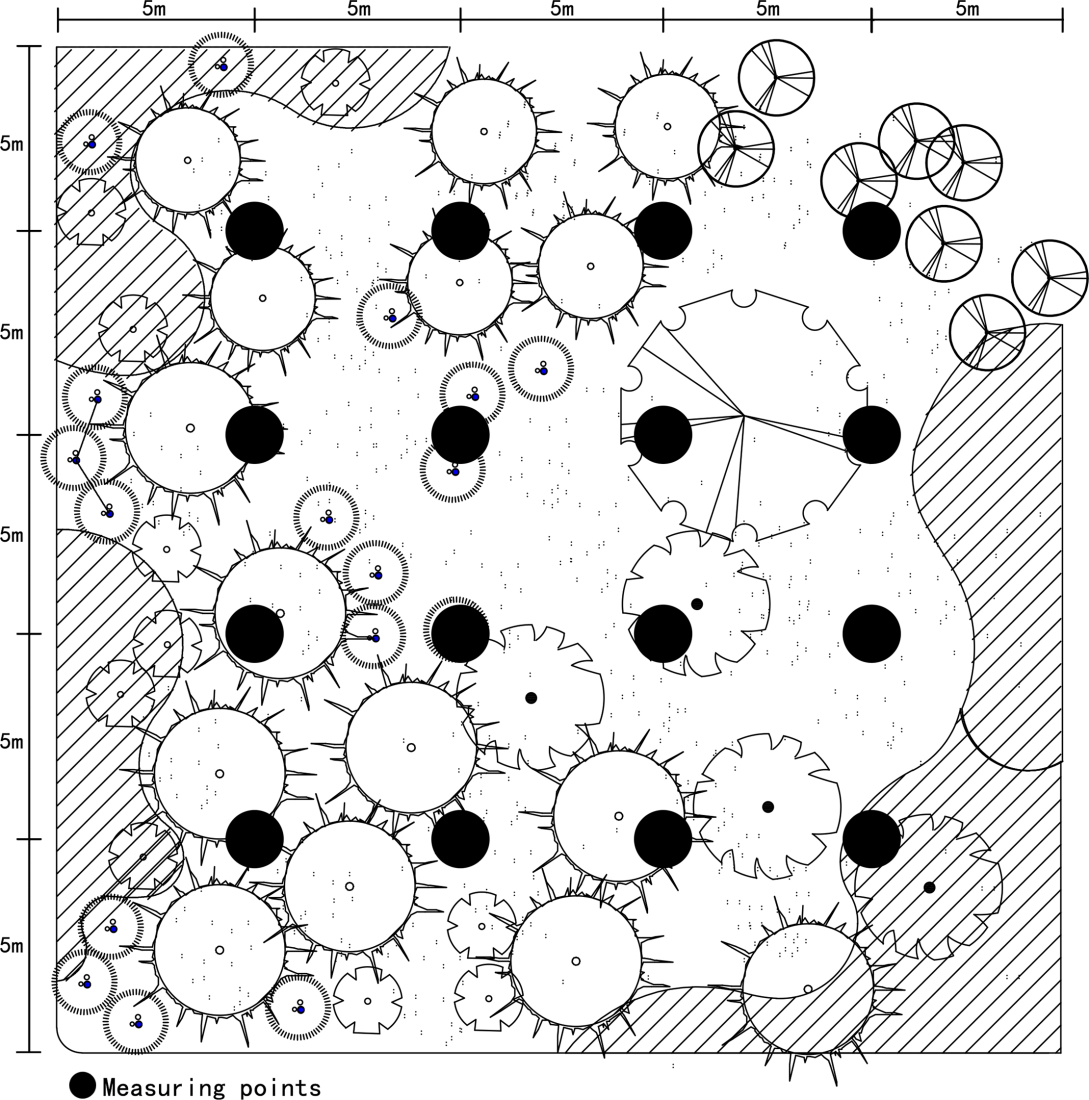
Location map of measuring points in one example plant community. The solid circles represent the measuring points where the structural characteristics, air temperature and relative humidity were measured.

During the field investigation of each plant community, the height (H, m) and diameter at breast height (DBH, cm) of each tree were measured and recorded. The CC of trees and shrubs/woodland area in each plant community as well as their total canopy cover ratio (Total CC) were also measured and recorded (**Table 1**). Furthermore, the leaf area index (LAI) and the canopy density (fraction occupied by tree canopy of the overlying hemisphere, CD) of plant community were measured with point transects. And the measuring points in each plant community were established uniformly on 5 m intervals between every two measuring points (**Fig 2**). The LAI at each measuring point was measured with an LAI-2200 plant canopy analyzer (LI-COR, Lincoln, NE, USA). Simultaneously, the CD was measured using a Sony DSC-RX100M2 digital camera (Sony Corp., Tokyo, Japan) at the measuring points, where the camera lens remained horizontal at a height of 1.5 m from the ground. Adobe Photoshop CS5 (Adobe System Inc., San Jose, CA, USA) was then used to change each photo into a gray two-tone image and to tally the image pixels using the following calculation [35]:

CD=1−ba

where *a* and *b* are the number of hemisphere and gap pixels of the enclosed canopy district, respectively.

#### Measurement of air temperature and relative humidity

Air temperature and relative humidity measurements were taken at 08:00–09:00 h, 11:00–12:00 h, 14:00–15:00 h and 17:00–18:00 h (LST = GMT + 8) from July 27 to August 7, 2015. The air temperature and relative humidity were measured at the established points in each plant community and control plots in Xincheng Central Park respectively at a height of 1.5 m above the ground. For measurements in each plant community, a corresponding measurement was made in the control plots. The measurements were conducted with a model NK5922 portable weather station (Kestrel, Boothwyn, PA, USA) when the wind speed fell below 2.0 m/s [10]. All measurements were repeated three times on different dates but with similar weather conditions. Based on the weather report from Shanghai Meteorological Bureau and our field investigation, sampling days were all calm (winds generally less than 2 m per sec) and sunny (less than 40% cloud cover) with air temperatures between 32–38 °C.

#### Questionnaire survey

The questionnaire mainly consisted of two parts: demographic variables (**Table 2**), and an evaluation of 15 color photographs of scenes with various scenes of plant communities with three different vegetation densities taken from various community parks in Shanghai. One aim of the questionnaire survey was to identify the potential plant community scenes that met respondents’ environmental perceptions for outdoor recreation in a community park. The questionnaires were delivered randomly to visitors in Xincheng Central Park during the field measurements from July 27 to August 7 (excluding rainy days) in 2015. The questionnaire survey was carried out during 7:00 to 9:00 am and 5:00 to 7:00 pm, when relatively large numbers visitors could be expected on hot summer days. Respondents were asked to evaluate each of the 15 photos representing the three vegetation densities and consider their appropriateness for recreation; photos were scored with a Likert five-point rating scale from 1 (not appropriate for recreation) to 5 (absolutely appropriate for recreation). In total, 128 questionnaires were delivered and collected on site.

**Table 2:** Data on the demographic variables of respondents provided in the questionnaires including gender, age, dwelling location, education level, and household size.

Characteristics	*N*	Ratio (%)	Characteristics	*N*	Ratio (%)
**Gender**			**Education level**		
Male	73	57.0	Primary and junior middle school	20	15.6
Female	55	43.0	High school and technical school	35	27.3
**Age**			Junior college and bachelor’s degree	60	46.9
<25 years	12	9.4	Graduate degree and above	13	10.2
25 years to 40 years	78	60.9	**Household size**		
40 years to 60 years	15	11.7	1 person	11	8.6
>60 years	23	18.0	2 people	21	16.4
**Dwelling location**			3 people	52	40.6
Central city	61	48.0	4 people	25	19.5
Urban suburb	44	34.3	≥5 people	19	14.8
Suburb area	23	17.7			

Note: *N*, sample size.

### Data analysis

Firstly, the vegetation densities in plant communities were identified and categorized based on the field investigation. Subsequently, paired-sample *T* tests were performed between plant communities and control plots to determine the cooling and humidifying effects of plant communities during each measuring period. The mean values of the cooling and humidifying effects from each plant community were figured out. One-way analysis of variance was generated to examine the significance of the average cooling and humidifying effects from the the open, moderately dense and dense plant communities. Significant analyses of variance (ANOVAs; *p*<0.05) were followed by Duncan’s multiple comparison tests (*p*<0.05) to separate the means of the three plant community types. In addition, Pearson correlation matrix and linear regression were used to analyze and quantify the relative contributions of the structural characteristics of plant communities to temperature reduction (ΔT) and humidity increase (ΔRH). Correlation analysis was conducted separately for each measurement period with the data of each measurement day, and the regression analysis was done with the averaged data of each measurement day. For the survey data from the questionnaire, respondents’ perceptions on plant community scenes with three different vegetation densities were evaluated. And one-way ANOVA was performed for analyzing the relationship between plant community preferences and the characteristics of respondents. All these statistical analyses were performed using the SPSS software package (version 18.0, SPSS Inc.).

## Results

### Vegetation densities in plant communities

**Fig 3:**
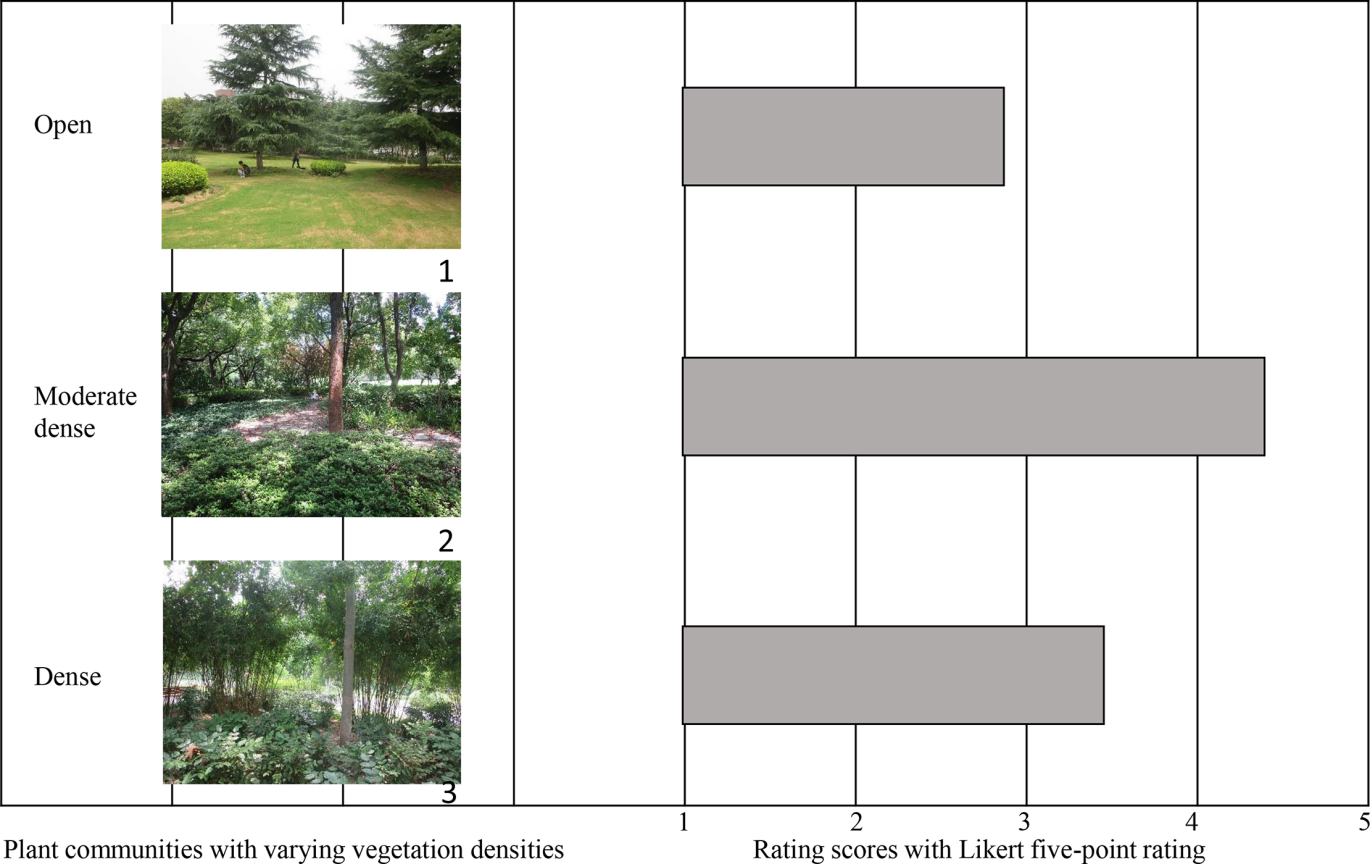
Photos of three representative plant community scenes with differences in vegetation density and rating scores for recreation. Photo 1 to 3 represented the open, moderately dense and dense plant communities, respectively.

Based on the field investigation, vegetation densities in plant communities were identified and categorized (**Table 1, Fig 3**). Within this gradient from open to dense, the herbaceous layer of vegetation varied from an urban park lawn to a natural herbaceous layer of vegetation with grass and herbs. The shrub layer varied from being absent to a dense layer of natural shrubs and bushes. The tree layers varied from scattered individual trees in open areas to a dense mix of trees with different sizes. In plant communities with open vegetation density, people could enter the site for recreation and the views of the surrounding landscape were essentially not blocked at all. On the contrary, people could not penetrate the plant communities with dense vegetation, and the vegetation physically blocked views of the surrounding landscape.

Furthermore, the canopy cover ratio of trees and shrubs/woodland area as well as their total canopy cover ratio in plant communities with varying vegetation densities were identified. For the open plant community, the total CC was <0.80, with <0.60 for trees and <0.20 for shrubs/woodland area. For the moderately dense plant community, the total CC was 0.80–1.20, with trees at 0.60–0.75 and the shrubs/woodland area at 0.20–0.45. Meanwhile, for the dense plant community, the total CC was >1.20, of this trees had >0.75 and shrubs/woodland area had >0.45.

### Cooling effects from plant communities

**Fig 4:**
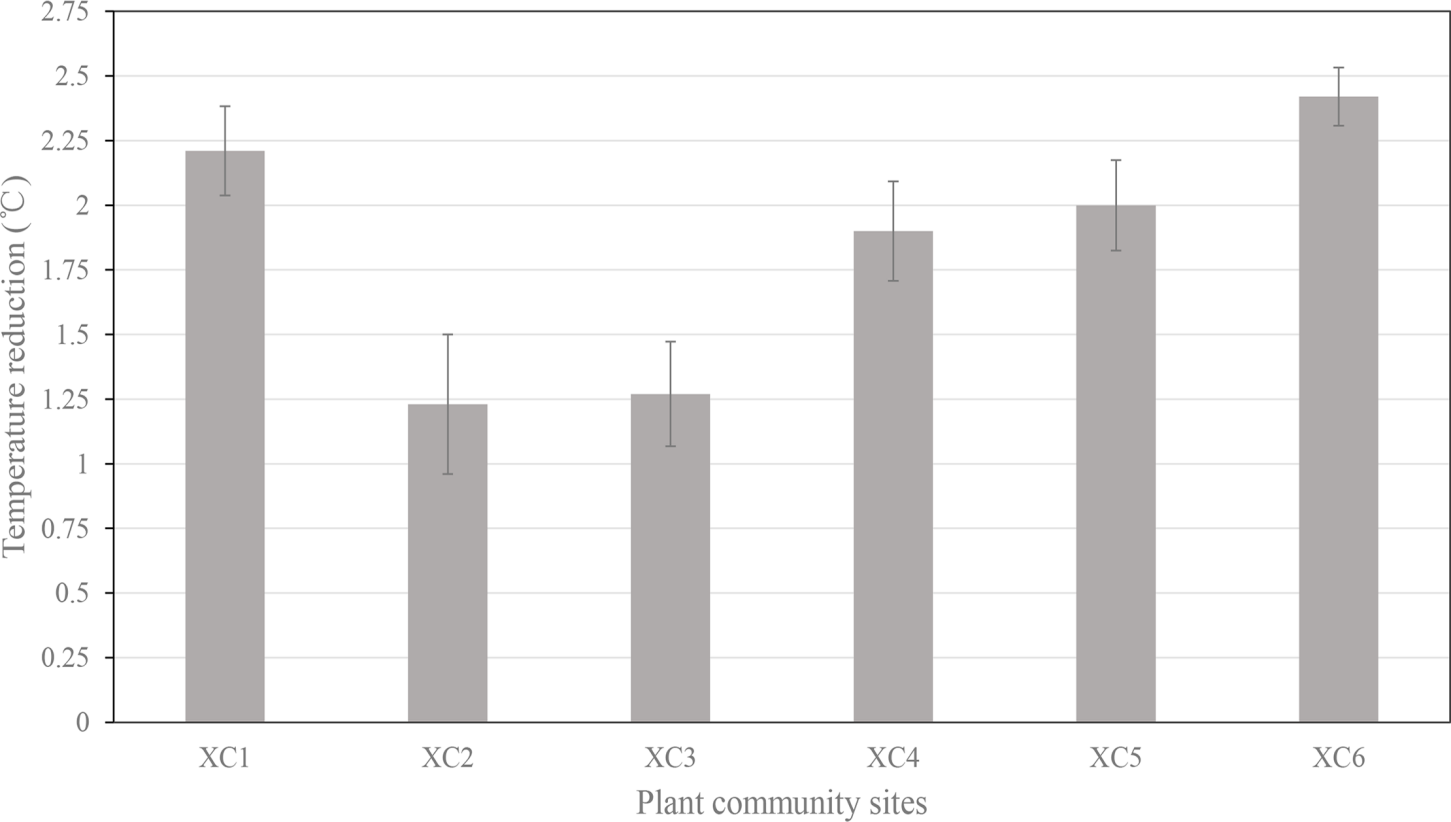
Mean value of diurnal temperature reduction in the plant communities. XC1 to XC6 are plant community site numbers in Xincheng Central Park.

Air temperatures in the control plots ranged from 33.82±0.24 °C to 37.16±0.21 °C, and within the plant communities, the temperature ranged from 32.02±0.08 °C to 35.04±0.41 °C. Based on the results of paired-sample *T* tests, air temperatures in plant communities were statistically lower than that of tree-free control plots during the measuring time, especially at 14:00–15:00 (**Table 3)**. The presence of plant communities resulted in a decrease in the air temperature by an average of 1.83 °C, and this decrease ranged from 1.23 °C in XC2 to 2.42 °C in XC6 (**Fig 4**).

**Table 3:** Mean air temperature and relative humidity based on the *T* tests between the plant communities and the control plots at different periods of daytime.

	T_8:00–12:00_ (°C)	T_11:00–12:00_ (°C)	T_14:00–15:00_ (°C)	T_17:00–18:00_ (°C)	RH_8:00–9:00_ (%)	RH_11:00–12:00_ (%)	RH_14:00–15:00_ (%)	RH_17:00–18:00_ (%)
**XC1**	32.04±0.19**	33.95±0.31**	34.32±0.38**	33.67±0.21**	71.5±1.4**	52.4±0.6**	48.1±1.2**	52.8±1.7**
**XC2**	33.04±0.45	35.03±0.26	35.04±0.41**	34.80±0.36	70.3±2.3*	51.6±1.1*	46.9±1.5	51.8±1.0*
**XC3**	33.07±0.37	34.93±0.25	34.94±0.35**	34.79±0.24*	69.8±2.6*	50.5±1.2	47.4±1.2	51.7±1.0
**XC4**	32.37±0.26	34.08±0.25**	34.71±0.35**	34.07±0.31**	72.7±0.6**	52.7±0.6**	48.0±1.3**	52.9±1.5**
**XC5**	32.28±0.23	34.03±0.29*	34.54±0.37**	33.98±0.21**	72.5±0.7**	53.0±0.8**	48.1±1.9**	53.1±0.7**
**XC6**	32.02±0.18**	33.6±0.26**	33.82±0.27**	33.74±0.14**	70.7±1.6**	52.0±0.8**	47.7±0.7*	52.8±1.6**
**CK**	33.82±0.24	36.24±0.10	37.16±0.21	35.63±0.25	66.2±2.5	48.0±0.8	45.6±0.4	50.1±0.5

Note: CK, control plots; RH, relative humidity; T, air temperature; XC1 to XC6, plant community site numbers in Xincheng Central Park.

* and ** indicate a significant air temperature difference between the plant community site and control plots at *p*<0.05 and *p*<0.001 respectively (2-tailed *T* tests).

A further ANOVA revealed that there were significant differences for the air temperature reductions from plant communities with varying vegetation densities during the daytime (*p*<0.05). Moderately dense plant communities (XC1 & XC6) were the most effective, with average temperature reductions of 2.31 °C. Dense plant communities (XC4 & XC5) and open plant communities (XC2 & XC3) reduced the temperature by 1.95 °C and 1.25 °C, respectively. Plant communities with open structures were the least effective at reducing summer temperatures. The cooling effects from open plant communities were quite different with the moderately dense (*p* = 0.002) and dense plant communities (*p* = 0.005), while the difference in cooling effects between moderately dense and dense plant communities was not significant (*p* = 0.118).

### Humidifying effects from plant communities

**Fig 5:**
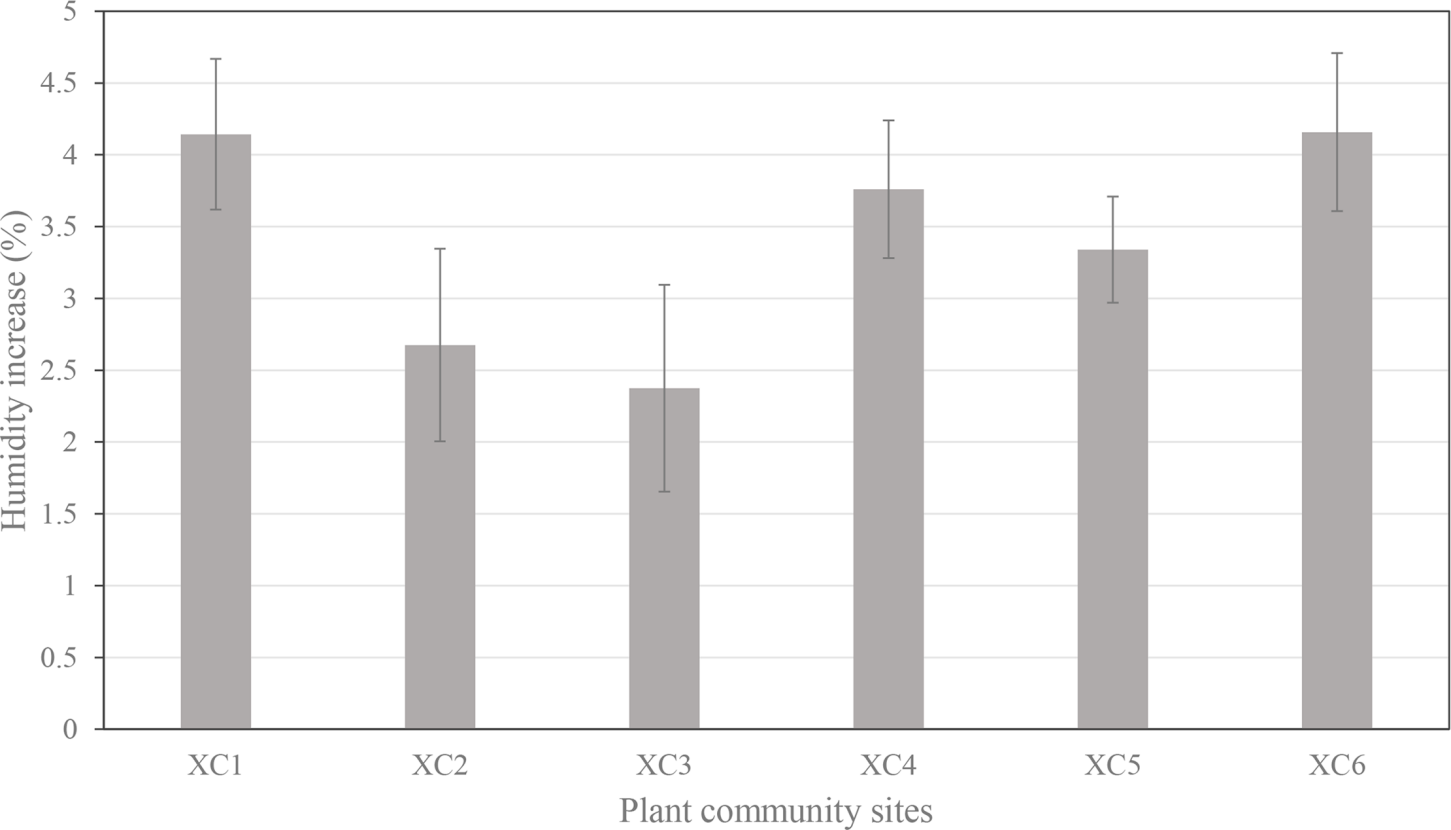
Mean value of diurnal humidity increase in the plant communities. XC1 to XC6 are plant community site numbers in Xincheng Central Park.

The relative humidity in the control plots ranged from 45.6±0.4% to 66.2±2.5%, and within the plant communities, it ranged from 46.9±1.5% to 72.7±0.6%. Based on the results of paired-sample *T* tests, the relative humidity was statistically higher in plant communities than in the tree-free control plots during the measuring time, especially at 8:00–9:00 (**Table 3**). The presence of plant communities resulted in an average 3.4% increase in relative humidity, and the increase ranged from 2.4% in XC3 to 4.2% in XC5 (**Fig 5**).

An analysis of variance was used to find the differences in the humidifying effects from plant communities with varying vegetation densities (ANOVA post hoc Duncan, all *p*<0.05). The results suggested that the presence of dense plant communities (XC4 & XC5) with more complicated structures resulted in the highest increase in relative humidity (4.2%). Meanwhile, plant communities with open structures (XC2 & XC3) had the lowest humidifying effects (2.5%). The humidifying effect from open plant communities was significantly different from moderately dense (*p* = 0.034) and dense (*p* = 0.009) plant communities. Additionally, significant difference in the humidifying effects was not found between moderately dense (XC1 & XC6) and dense (XC4 & XC5) plant communities (*p* = 0.127).

### Factors affecting the cooling and humidifying effects from plant communities

To clarify the cooling and humidifying effects from plant communities, Pearson correlation analysis was undertaken to examine the relationship between plant community structural characteristics and cooling as well as humidifying effects with regards to the measuring time (**Table 4**). It was found that the temperature reduction caused by the presence of plant communities had a significant positive correlation with LAI (*R* = 0.783, *p* = 0.033) and CD (*R* = 0.753, *p* = 0.042), while the increase in the humidity of plant communities had a significant positive correlation with CD (*R* = 0.876, *p* = 0.011) and total CC (*R* = 0.760, *p* = 0.04). In addition, these correlations may change during the daytime. Over the course of the measuring periods, LAI was found more often statistically significant for determining the cooling effect. While for the humidifying effect, the correlation with CD was more often statistically important. For the other structural characteristics of plant communities, no significant correlations with temperature reduction and humidity increase were found.

**Table 4:** Person correlation coefficients between the structural characteristics of plant communities and temperature reduction (ΔT) as well as humidity increase (ΔRH).

Characteristics	Item	08:00–09:00	11:00–12:00	14:00–15:00	17:00–18:00	Daytime
**LAI**	ΔT	0.686	0.792*	0.862*	0.737*	0.783*
	ΔRH	0.667	0.544	0.778*	0.777*	0.716
**CD**	ΔT	0.689	0.724	0.809*	0.742*	0.753*
	ΔRH	0.779*	0.757*	0.911**	0.955**	0.876*
**Total CC**	ΔT	0.398	0.419	0.555	0.463	0.464
	ΔRH	0.587	0.720	0.757*	0.930**	0.760*

Note: Total CC, total canopy cover ratio; CD, canopy density; LAI, leaf area index

* and ** indicate significance at *p*<0.05 and *p*<0.001, respectively.

**Fig 6:**
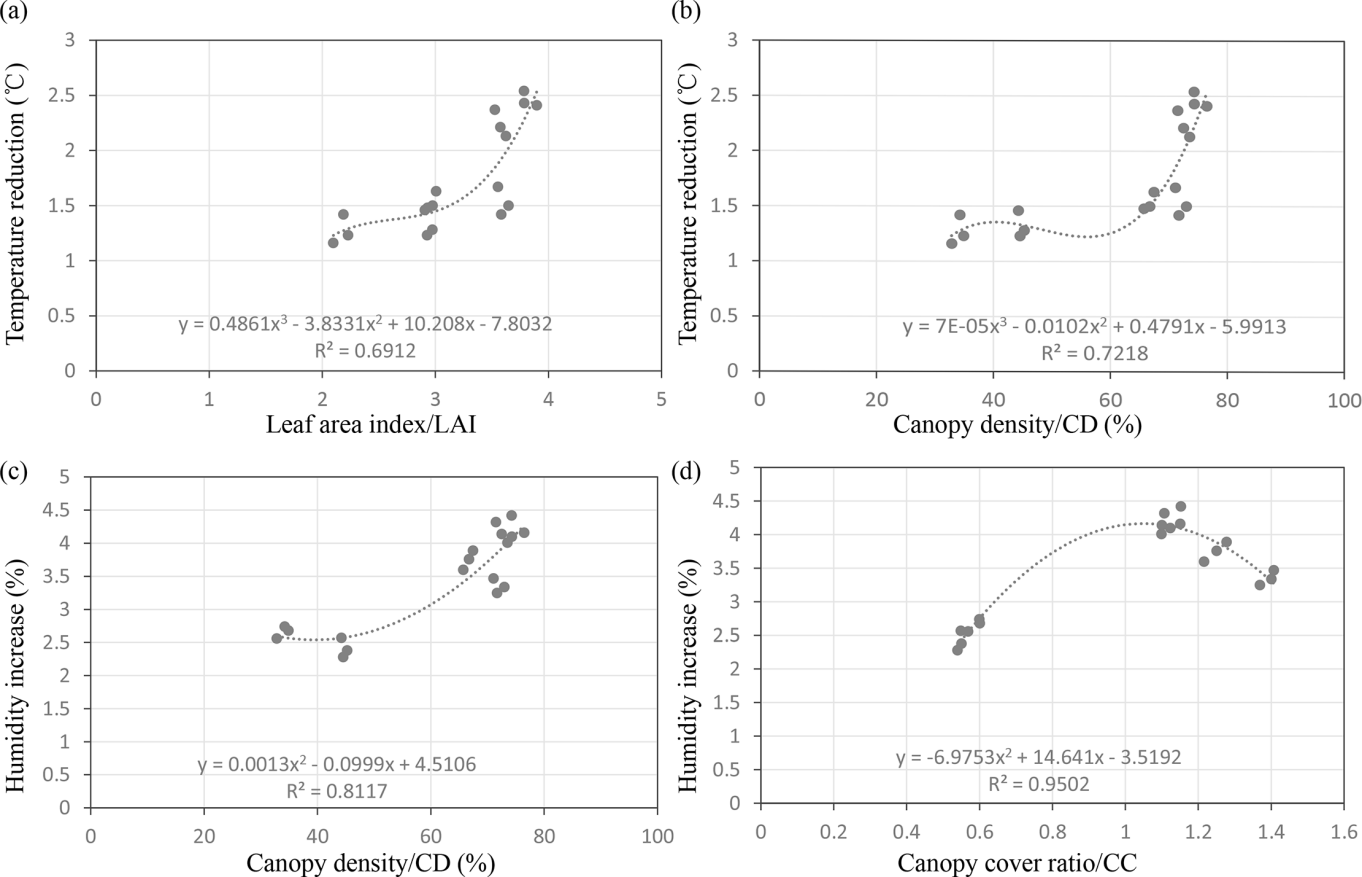
Regression analysis between the structural characteristics and temperature reduction as well as humidity increase.

When considering the structural characteristics that correlated with the cooling and humidifying effects of the plant community, linear regression analysis was used to quantify the relationship between the cooling effect, LAI and CD as well as between the humidifying effect, CD and total CC (**Fig 6**). The temperature reduction seemed to increase unevenly with increases in LAI and CD. Specifically, the incremental rate of temperature reduction was much higher when the LAI was above 3 and the CD was above 60%. Meanwhile, for the increase in humidity, the increment was much higher when the CD was above 50% and the total CC was close to 1.

### Appropriateness for recreation of different plant community densities based on the questionnaire

According to the results of questionnaire survey, respondents’ perceptions on the plant community scenes with three different vegetation densities could be found in **Fig 3**. The landscape scene portrayed in Photo 2, which was representative for the moderately dense plant community, was preferred by most respondents for outdoor recreation in park areas. That is, the mean values of moderately dense plant community (4.35) were significantly higher (p<0.05) than that of open (2.83) and dense (3.47) plant community scenes. By further investigating the plant community scenes that met most respondents’ environmental perceptions, it could be found that these plant communities could not only contain moderately dense vegetation but also provide some spaces for outdoor recreation.

**Fig 7:**
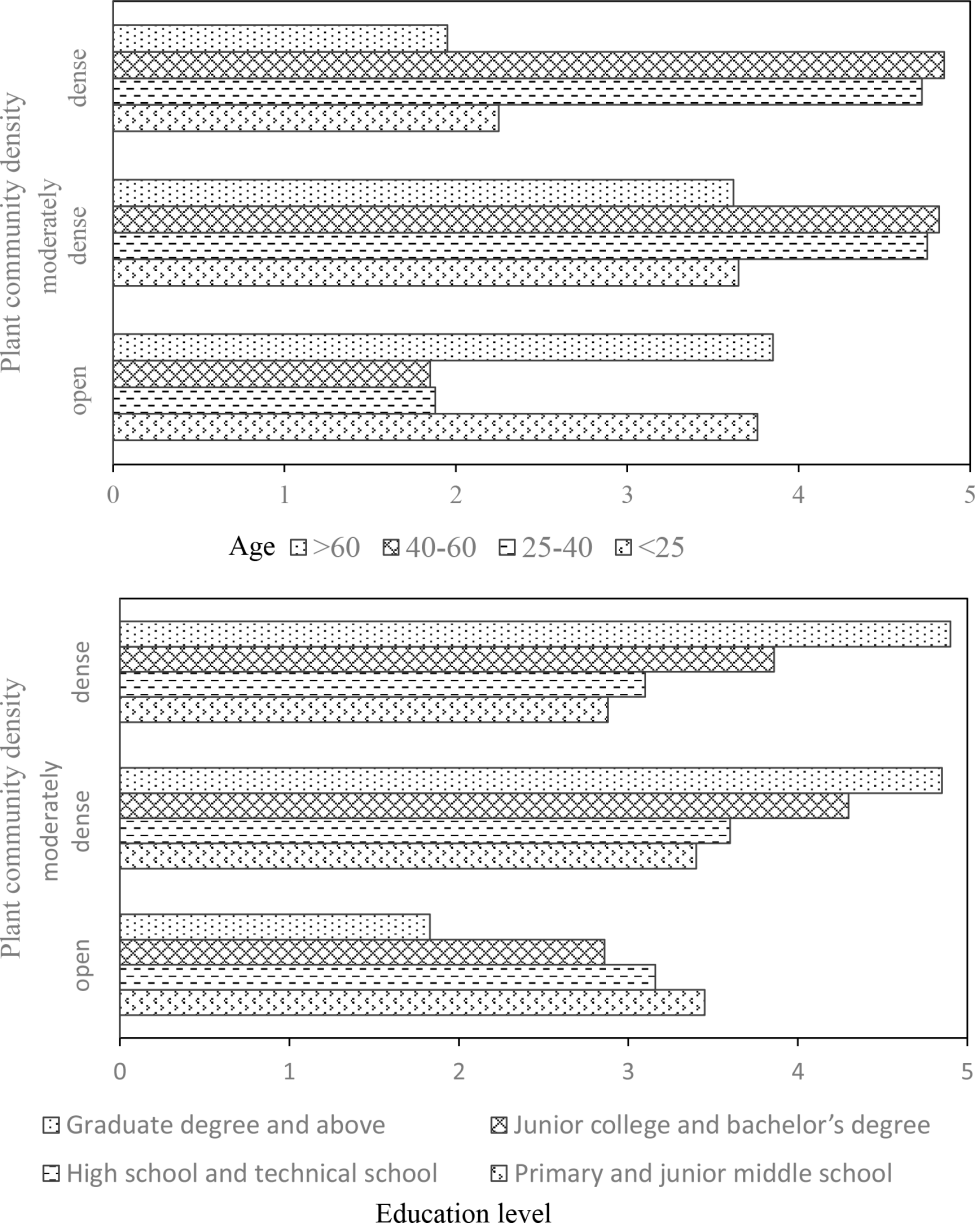
Preference on the plant community density of respondents with different ages and education levels.

Meanwhile, the open and dense plant communities in parks were also assessed as favorable for recreation to some extent (**Fig 3**). One-way analysis of variance (ANOVA) found that demographic variables of the respondents would influence their perceptions on plant community density (**Table 5**). The results revealed that the age and education level of these respondents had a close relationship with this assessment (*p*<0.05). Respondents age 40–60 and 25–40 expressed a higher preference for moderate to dense vegetation density, compared with both younger and older subjects. The respondents age < 25 and >60 generally preferred the open plant communities which could provide the highest accessibility for recreation. For the education level, respondents with higher education level would prefer plant community scenes from moderate to dense vegetation density possibly due to their knowledge of the benefits from dense vegetation (**Fig 7**).

**Table 5:** Analysis of variance of respondents’ preference for plant community densities in relation to their demographic characteristics.

Characteristics	Df	f	*p*
**Gender**	1	20.845	0.078
**Age**	3	29.723	0.000
**Education level**	3	25.551	0.013
**Household size**	4	20.523	0.064
**Dwelling location**	2	24.823	0.167

## Discussion

### Structural characteristics of plant community related with the cooling and humidifying effects

Although many previous studies have investigated the cooling and humidifying effects of plant communities [13, 36, 37], these previous studies have concentrated mainly on the diurnal averages of meteorological conditions and neglected their moment-to-moment changes. The present study clarified that the structural characteristics of plant communities were related to the cooling and humidifying effects at any period of a sunny summer day and further found that the correlations between them appeared to change over time (**Table 4**). A possible explanation for this finding on the cooling effect is that the interception of the tree canopy is diverse because of the incident angle of radiation changes throughout the day. When the angle is low and its intensity is weak, the cooling effect seems to largely depend on the CD. Whereas, the cooling effect may be more controlled by both CD and LAI when the angle slowly increases [21]. However, when compared with air temperature, relative humidity is not only similarly influenced by vegetation structural features but is also variously influenced by transpiration, soil moisture, irrigation time, and soil evaporation [38], which are not considered in the present study. Even though the changes in the correlations over time are new findings, a considerable amount of confusion remains to be resolved such as the varying canopy characteristics from different tree species. The selection of some common monodominant communities and conducting studies regarding this topic over much shorter time scales may help us to further understand the influence of vegetation structural characteristics on cooling and humidifying effects of plant communities.

In the present study, it was found that the microclimate conditions in plant communities with varying vegetation densities were significantly different. Furthermore, the study verified that both CD and LAI were better indicators than total CC (vegetation density) for predicting the cooling effects from plant communities. This result is consistent with the findings of Peters and McFadden in which the temperatures of plant communities were mainly controlled by differences in the stand-level LAI and CD [39]. Most likely, the air temperature within a plant community is most affected by beams of solar radiation penetrating the canopy, which is consistent with studies that have discussed the diurnal variation and primary drivers of the cooling effect in urban green spaces [13, 37, 40]. Meanwhile, both CD and total CC were significant indicators of the humidifying effects from plant communities, meaning that the complicated structure with some understory shrubs would increase the humidity in plant communities. The theory for this finding could be explained as follows. First, a complicated vegetation structure could result in more water being released through physiological processes [41, 42]. Moreover, the high CD could prevent water from being lost from the vicinity of the vegetation that produces it [43, 44].

### Recommendation for plant communities with appropriate cooling and humidifying effects for recreation

Plant communities in community parks are not only developed for their environmental benefits but are also designed to attract more visitors for recreation. According to many previous studies, plant community density is considered one of a number of environmental features contributing to the perceived appropriateness for outdoor recreation in park areas. Based on the opinions obtained through use of a questionnaire, the plant community landscapes that varied in three vegetation densities were all perceived as favorable for recreation, while the moderately dense scenes received the highest ratings. This finding confirmed some previous studies that defined the role of openness as an important environmental attribute of parkland and showed that moderately dense scenes were generally preferred by most respondents [27, 34]. People could enter into these plant community sites without obstructions for contacting with nature [23, 45]. Furthermore, respondents with varying ages or education levels may have different perceptions on the appropriateness of various plant community densities due to their personal locomotion abilities and special demands in parks. These results are also in accordance with some previous studies showing that demographic variables of respondents may predict preferences for different plant communities [29]. In the present study, high preferences for the moderately dense plant community may be attributed to the fact that about 60.9% (**Table 2**) of the respondents had the age of 25–40 who expressed a higher preference for moderate to dense vegetation density.

Given the results above, some recommendations could be given for the landscape design methods used in community parks. The plant communities within a park should harmonize the functions of mitigating the thermal environment with the environmental perceptions most people have of the preferred vegetation density. First, temperature reduction from plant communities was mainly controlled by differences in the stand-level LAI and CD. The CD should be no less than 60% and LAI should be no less than 3 to ensure plant communities provide the desired cooling effects. And the CC of trees should be no less than 0.60 to ensure the appropriate CD (60%) of one plant community. Further considering the environmental preference of most respondents for moderately dense plant community, the CC of trees should be 0.60–0.75 while the CC of shrubs/woodland area should be 0.20–0.45 (total CC 0.80–1.20). This type of plant community pattern could not only improve the thermal environment, but also meet the perceptions and demands of most respondents while providing recreation opportunities in a park. However, there are still some drawbacks in the recommended plant community pattern which should be further studied in the later research. Firstly, respondents with varying demographic variables may have different perceptions on plant community density and the respondents in the present study mainly had the age of 25–40, thus different plant community patterns should be further studied and developed for varying visitors in community parks. Furthermore, the present study mainly focused on the structural characteristics of plant communities without considering their species composition, which could influence the visual landscape [46] and climate control services [47] of plant communities.

## Conclusions

The results presented in this study clearly showed that plant communities in community parks had an obvious potential to mitigate heat stress in hot summers, although the effects varied in different plant communities and time periods. Compared to the control plots, plant communities could reduce the air temperature slightly and increase the relative humidity. The temperature reduction from the plant community was most significant at 14:00–15:00, and the humidity increase was most significant at 8:00–9:00. Furthermore, our results indicated that even the microclimate conditions in plant communities varying in vegetation density were significantly different, both CD and LAI were better indicators than total CC for predicting the cooling effects from plant communities. The CD and total CC were effective in increasing the relative humidity. This study further clarified that these correlations appeared to change over time.

In addition, moderately dense plant communities with some space for recreation in park areas were perceived most appropriate for outdoor recreation by respondents based on our questionnaire. And the demographic variables of respondents would have impacts on their perceptions of plant community density. All these survey results have important implications for the planting and landscape designs in community parks. This study suggests that adjusting the structural characteristics of plant communities based on the contributions of those communities to cooling and humidifying effects and simultaneously balancing the nature of the plant communities with the residents’ perceptions of the desired level of vegetation density required for recreation would facilitate the adoption of an improved planting design. Plant communities with appropriate canopy density (≥60%), leaf area index (≥3) and canopy cover ratio (total 0.80–1.20, 0.60–0.75 for trees and 0.25–0.4 for shrubs/woodland area) were recommended in parks, that would harmonize the functions of the mitigation of the thermal environment with most people’s perceptions of a desirable vegetation density for outdoor recreation.

## Supporting information

S1 File Copyright statement from the National Geographic Information Public Service Platform of China. (PDF)

S2 File Questionnaire used to identify plant communities that were preferred by respondents with regard to recreation in a park. (PDF)
