# Supplementary material for: The ecological services of plant communities in parks for climate control and recreation—A case study in Shanghai, China
Source: PLoS One. 2018 Apr 25;13(4):e0196445. doi: 10.1371/journal.pone.0196445 (PMC5919075; doi:10.1371/journal.pone.0196445)
Supplement: S1 File — (PDF) [file pone.0196445.s001.pdf]

Copyright Statement from the National Geographic Information Public Service Platform of China

1. The website of this platform is http://www.tianditu.com/. And the printScreen of this website was shown as following:

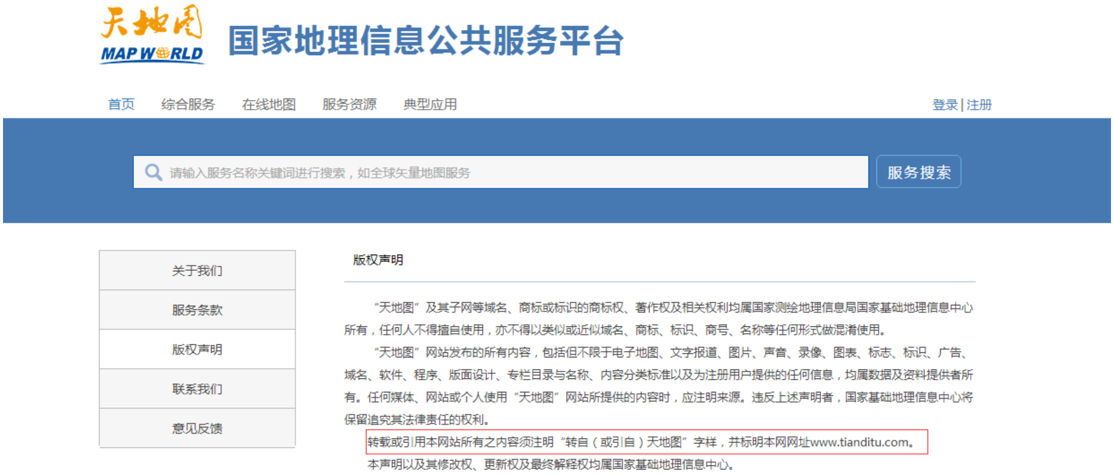

2. Copyright Statement

This is one public service platform of China, and thus the language in this website is Chinese.

版权声明 (Copyright Statement)

“天地图”及其子网等域名、商标或标识的商标权、著作权及相关权利均属国家测绘地理信息局国家基础地理信息中心所有，任何人不得擅自使用，亦不得以类似或近似域名、商标、标识、商号、名称等任何形式做混淆使用。

“天地图”网站发布的所有内容，包括但不限于电子地图、文字报道、图片、声音、录像、图表、标志、标识、广告、域名、软件、程序、版面设计、专栏目录与名称、内容分类标准以及为注册用户提供的任何信息，均属数据及资料提供者所有。任何媒体、网站或个人使用“天地图”网站所提供的内容时，应注明来源。违反上述声明者，国家基础地理信息中心将保留追究其法律责任的权利。

转载或引用本网站所有之内容须注明“转自（或引自）天地图”字样，并标明本网网址www.tianditu.com。

本声明以及其修改权、更新权及最终解释权均属国家基础地理信息中心。

According to the the copyright statement from the National Geographic Information Public Service Platform of China, any media, websites or individuals could use or reprinting the data from this platform with indicating the datasource (**MAPWORLD**) and website (**www.tianditu.com**).
