# Supplementary material for: The ecological services of plant communities in parks for climate control and recreation—A case study in Shanghai, China
Source: PLoS One. 2018 Apr 25;13(4):e0196445. doi: 10.1371/journal.pone.0196445 (PMC5919075; doi:10.1371/journal.pone.0196445)
Supplement: S2 File — (PDF) [file pone.0196445.s002.pdf]

Questionnaire used to identify plant communities that were preferred by respondents with regard to recreation in a park.

The following questionnaire is an English translation of the Chinese language questionnaire used in this study. Each respondent was given a paper copy of the questionnaire and asked to respond. Here is the questionnaire:

This survey uses the following questionnaire to identify the plant communities that respondents prefer to use for recreation in parks in Shanghai. The questionnaire will ask for 15 responses. Participation is completely anonymous and voluntary. The data collected from the questionnaire will only be used for academic research.
Many thanks for your participation in this survey.

Survey site ____________Survey time: _________

I understand the contents and purpose of this survey. I would like participate in the survey and provide the information related to the questionnaire anonymously.
① Agree ② Disagree

Please choose one for each item:

(1) Gender: ① Male ② Female

(2) Age: ① <18 ② 18–44 ③ 45–60 ④ >60

(3) Education level:

① Primary and junior middle school ② High school and technical school

③ Junior college and bachelor's degree ④ Graduate degree and above

(4) Household size: ① 1 ② 2 ③ 3 ④ 4 ⑤ ≥5

(5) Dwelling location: ① city center ② city suburbs ③ suburbs

(6) Considering the appropriateness for recreation, please rank the pictures of each plant community in the following table from 1 (not appropriate for recreation) to 5 (very appropriate for recreation)

Community type	Picture	Rating of appropriateness for recreation				
Open	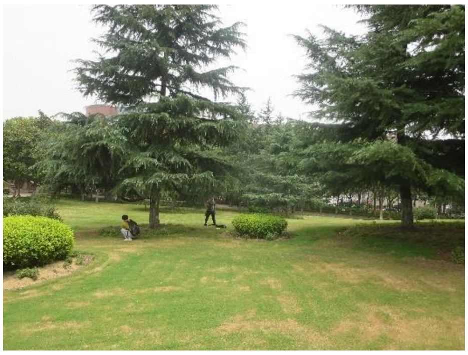	1	2	3	4	5

	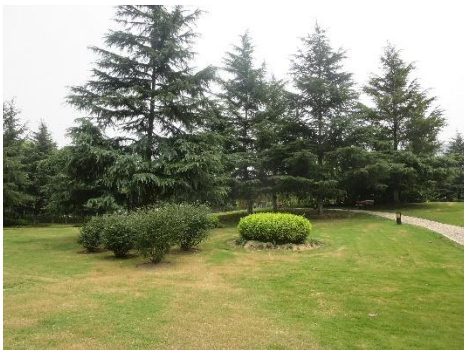	1	2	3	4	5
	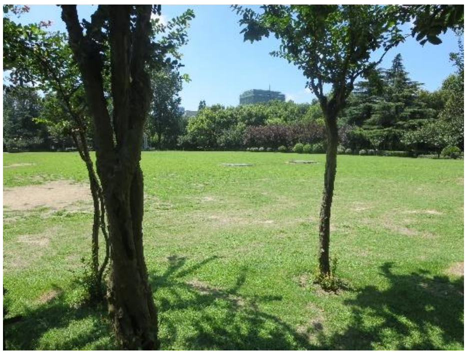	1	2	3	4	5
	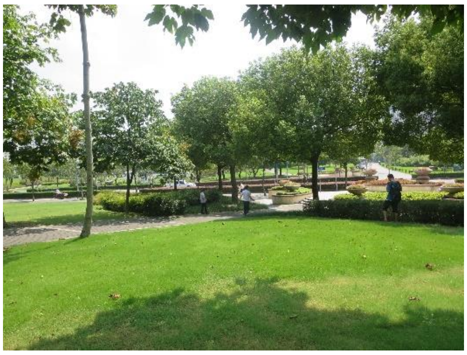	1	2	3	4	5
	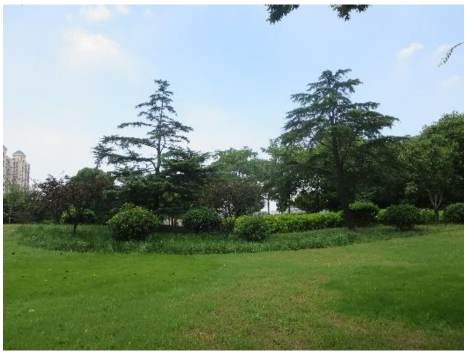	1	2	3	4	5

Moderately open	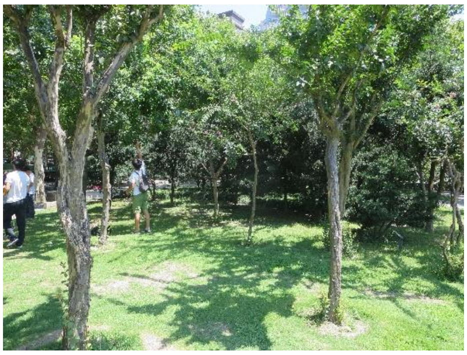	1	2	3	4	5
	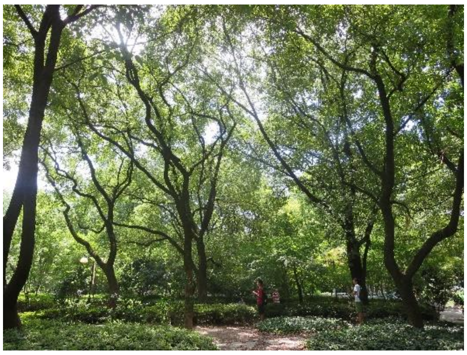	1	2	3	4	5
	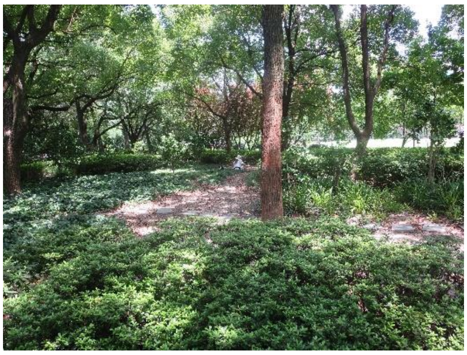	1	2	3	4	5
	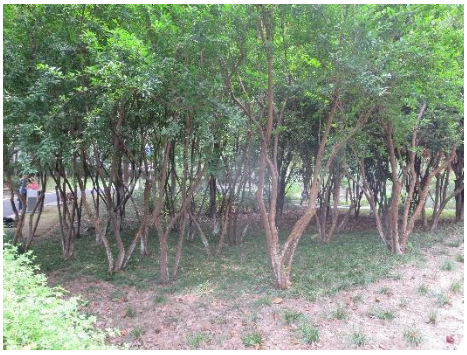	1	2	3	4	5

	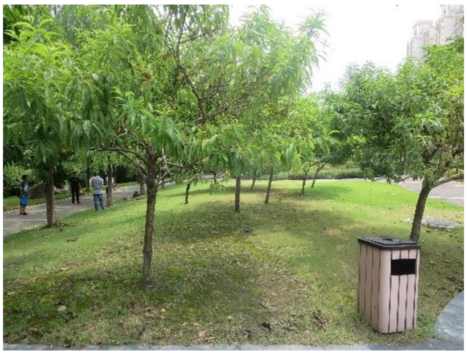	1	2	3	4	5
Dense	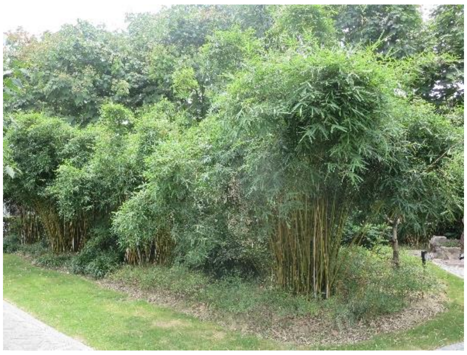	1	2	3	4	5
	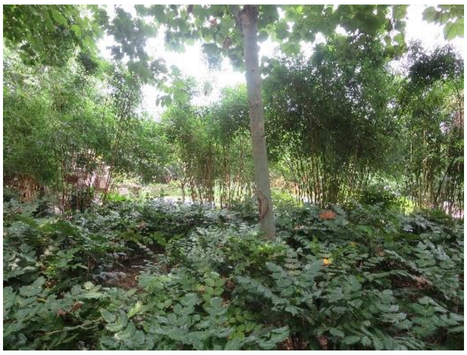	1	2	3	4	5
	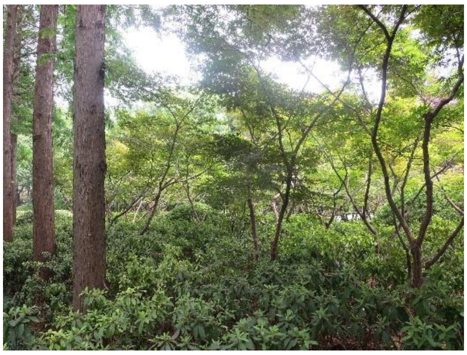	1	2	3	4	5

	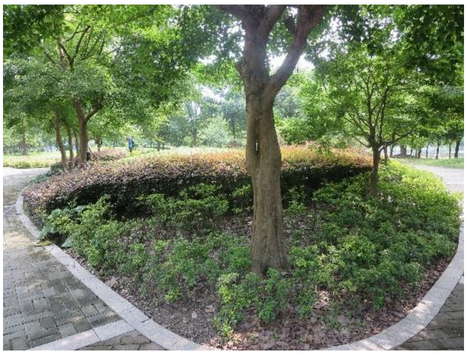	1	2	3	4	5
	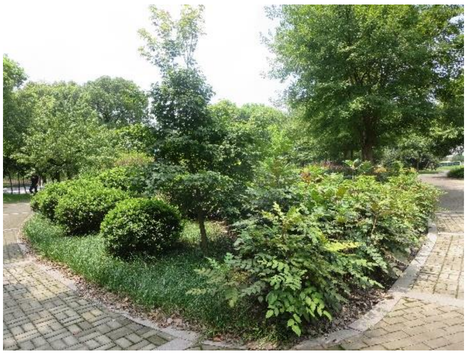	1	2	3	4	5

This is the end of the questionnaire!
Thank you again for your support and cooperation!
